# Longitudinal study on background lesions in broiler breeder flocks and their progeny, and genomic characterisation of *Escherichia coli*

**DOI:** 10.1186/s13567-022-01064-7

**Published:** 2022-07-07

**Authors:** Sofie Kromann, Sharmin Baig, Marc Stegger, Rikke Heidemann Olsen, Anders Miki Bojesen, Henrik Elvang Jensen, Ida Thøfner

**Affiliations:** 1 Department of Veterinary and Animal Sciences, Faculty of Health and Medical Sciences, University of Copenhagen, 1870 Frederiksberg, Denmark; 2 DanHatch Denmark A/S, Rugerivej 26, 9760 Vrå, Denmark; 3 Department of Bacteria, Parasites and Fungi, Statens Serum Institut, Artillerivej 5, 2300 Copenhagen, Denmark

**Keywords:** Mortality, pathology, antimicrobial resistance, *Escherichia coli*, colibacillosis, whole-genome sequencing, APEC, avian pathogenic *E. coli*, surveillance

## Abstract

In broiler breeders, background mortality is rarely addressed, however, it represents the death of a vast number of birds, a constant productivity loss, welfare concerns and it might affect chick quality. The study aimed to unveil lesions leading to mortality in a study population perceived as healthy, combined with whole-genome sequencing (WGS) of *Escherichia coli*, a well-known contributor to disease problems in poultry. Broiler breeders (*n* = 340) originating from three distinct, putative healthy flocks and their progeny (*n* = 154) were subjected to a comprehensive post-mortem examination, bacteriological sampling, and sequencing of 77 *E. coli* isolates. Productivity data confirmed an exemplary health status of the enrolled flocks, and post-mortem examination further verified the absence of general disease problems. Among the submitted broiler breeders, exudative peritonitis (31.2%) was the most frequent lesion linked to infectious disease, whereas airsacculitis, pericarditis, perihepatitis, and salpingitis occurred in 18.5%, 3.5%, 3.8% and 17%, respectively. Yolksacculitis occurred in 15.6% of the broilers, whilst pericarditis, perihepatitis and peritonitis were diagnosed in 9.7%, 7.1% and 9.1%, respectively. WGS revealed a diverse population where ST95 dominated the population retrieved from broiler breeders, whereas ST10 was highly prevalent among broilers. Both lineages could be isolated from extraintestinal sites of birds without lesions indicative of infection. In general, the genetic diversity within flocks was comparable to the diversity between farms, and the overall occurrence of resistance markers was low. In conclusion, a comprehensive insight into lesions associated with background mortality is presented, together with a vast diversity of *E. coli* isolated from extraintestinal sites during a non-outbreak situation.

**Supplementary Information:**

The online version contains supplementary material available at 10.1186/s13567-022-01064-7.

## Introduction

In broiler breeders, a mortality as high as approximately 8% can be expected throughout a production period in non-outbreak situations [1, 2]—i.e., when only the “background” mortality contributes to death and culling. Despite this represents a vast number of birds, lowered productivity and animal welfare concerns, background mortality is only rarely addressed [1].

Contributing further to the importance of underlying disease problems in broiler breeders is the established link between the parent stock and the quality and health status of the broilers [2–4].

A well-known contributor to morbidity and mortality in poultry is *Escherichia coli*, which has for long been considered the most important bacterial pathogen in poultry production [5–7] being a leading cause not only of mortality (death/culling) but also the main cause of carcass condemnation in broilers and broiler breeders [8–11]. Colibacillosis in poultry has formerly been viewed as an opportunistic disease secondary to other factors such as viral or *Mycoplasma* infections*,* high ammonia levels or stress [5, 12, 13]. However, more recently it has been speculated that high-virulent *E. coli* strains may be primary pathogens in case of “true” avian pathogenic *E. coli* (APEC) [5, 14]. Efforts have been made to unveil the transmission and describe APEC in-depth, especially in situations with elevated *E. coli*-related problems and/or across wide timespans [15–17]. In recent years, high-resolution techniques, such as whole-genome sequencing (WGS), have been applied with success and the method holds great potential in the quest to portray and define the APEC pathotype [18, 19]. Yet, the ability to genomically define this pathotype has been highly obscured by the lack of differentiation between APEC-driven and opportunistic colibacillosis, thereby, rendering the term APEC almost meaningless [18]. Therefore, it is essential to view *E. coli*, isolated from lesions within poultry, in a larger context taking flock mortality, morbidity, productivity and, especially, necropsy findings into consideration when designating strains as APEC.

The current study aimed to unveil the overall pathology present in a study population unaffected by general disease problems, i.e., the lesions leading to background mortality, and further characterise the isolated *E. coli*-types through genomic analyses.

## Materials and methods

### Study design

From September 2019 to August 2020, a total of 360 birds were scheduled to be collected from three distinct Ross 308 broiler breeder farms (A, B and C). Appointment to the study was based on historical productivity data provided by DanHatch Denmark A/S and all flocks had adhered to a standardised vaccination program during rearing containing Poulvac *E coli*^®^ and an autogenous vaccine targeting *E. coli*. The selection criteria included cumulated mortality, disease history, and production data (e.g., hatchability, chick quality, farmer compliance etc.). From all farms, the birds were collected at two distinct stages of the production period, i.e., during week 27–34 and 45–60 of life, respectively. These time periods encompassed different yearly seasons, the farms were not synchronised, and each received birds originating from separate rearing farms. Farmers collected the birds following specific instruction from the field veterinarian, and euthanised birds were immediately placed at −20 °C until the time of necropsy. Cervical dislocation was applied in cases of euthanasia. Collection criteria were as follows: birds exhibiting depression, soiled cloacal areas, lacerations (mating injuries), lameness, or other clear signs of disease were gathered, and the field veterinarian visited the farms regularly during the study period. Birds with obvious lesions consistent with cannibalism and spontaneously dead birds with clear signs of cadaverosis were not collected. In the rare case that birds with any signs of cadaverosis were still submitted to the study, the birds were  excluded prior to necropsy due to a high probability of bacterial overgrowth and the inability to evaluate organs and identify lesions in such animals. From each collection period, the farmer was told to euthanise a minimum of 10 clinically healthy birds to serve as controls.

In addition to broiler breeders, progeny broilers (age 4–11 days) from each of the included parent farms, hatched from eggs laid during week 27–34 and showing signs of disease and/or weakness, were collected.

### Post-mortem examination

All birds were subjected to a thorough necropsy [20], and lesions were registered according to a standardised scheme (Additional files 1 and 2). Briefly, the surface of the animal was inspected, and lesions on skin, plumage and footpads were registered. The entire respiratory system, including sinus infraorbitalis, were examined on the surface as well as the larger luminal areas, content of the crop was noted, whilst the remaining intestinal tract was inspected on the surface and opened upon indication. However, in young progeny the ventriculus was routinely cut open and inspected. In broiler breeders, the salpinx was opened, and the reproductive status was evaluated with registration of the presence of a developing egg, fully developed egg, or absence of the former. Follicular status was also noted. The hip-, knee- and hock joints were opened and inspected, whilst other joints and parts of the locomotion system were examined in-depth upon indication. Musculus pectoralis superficialis and profundus were incised routinely for inspection. The kidneys were examined in situ, whilst the lungs, heart, liver, and spleen were removed from the carcass for inspection. In the broilers, the navel area, and the yolk sac, if still present, were carefully evaluated.

### Microbiology

From all broiler breeders, bacteriological sampling from the right lung, liver and salpinx was performed using sterile wooden cotton swabs or steel cotton swabs, depending on the organ sampled with subsequent streak-plating on blood agar base supplemented with 5% bovine blood. Additional samples were collected upon indication. Prior to sampling, the organ surfaces were sterilised with a burning hot iron spatula in order to reduce contamination during necropsy. The plates were incubated at 37 °C overnight and examined for growth. Tentative *E. coli* in pure growth were sub-cultured using MacConkey agar, and freeze stock was prepared for storage at −80 °C until further analysis. From the progeny, swabs were collected from the liver and yolk sac, or the liver and left lung if the yolk sac had been completely resorbed. Classification of isolate-origin into birds suffering from colibacillosis or not was based on nearly indisputable signs of this disease, i.e., presence of fibrinous, purulent or fibrinopurulent exudate [5], concurrent with the recovery of abundant pure growth of *E. coli*.

### Whole-genome sequencing

A subset of the collected *E. coli* isolates was selected for WGS with an aim of ensuring representation of multiple organs and birds from all farms. Genomic DNA was extracted using an enzymatic pre-lysis step before automated purification using the MagNA Pure 96 DNA and Viral NA Small Volume Kit and the DNA Blood ds SV 2.0 protocol (Roche Diagnostics) and quantified using the Qubit fluorometer (Invitrogen, Waltham, MA, USA). Subsequently, library construction and sequencing were performed using the Nextera XT Kit (Illumina, Little Chesterford, UK) and 300-cycle kits on the NextSeq 550 (Illumina) platform according to the manufacturer’s instructions. Quality control of the sequencing data was performed using bifrost [21] to ensure adequate sequencing depth, species verification and contamination issues of isolates prior to assembly using SPAdes v3.9.0.

For analysis into the relatedness of the isolates, the raw sequencing data were aligned to the joined contigs of *E. coli* E51 (GenBank accession number LYPJ00000000) [22] using NASP v1.0.0 [23] with BWA-MEM [24] and subsequent single nucleotide polymorphisms (SNPs) were called using GATK [25]. If a variant was present in <90% of the base calls per site across any individual isolate or a minimum coverage of 10 was not met, the position was excluded across the collection to retain only high-quality variant callings. The relatedness of the isolates was inferred utilising IQ-TREE v1.6.9 [26] using ModelFinder and bootstrap analysis using 100 replicates. The tree was mid-point rooted. For visualisation of the phylogeny and key genetic characteristics, iTol v6.4.2 was employed. Serotype prediction was performed in silico on assemblies utilising ECTyper v1.0 [27]. Sequence types (ST) were assigned using MLST [28] based on implementation of the Wirth et al*.* [29] typing scheme at PubMLST. To detect acquired resistance genes, ARIBA [30] was run on raw reads on the ResFinder database (accessed 5^th^ December 2021). Only hits with minimum 90% sequence similarity and 90% coverage of the reference genes were considered present. Additionally, PointFinder [31] was used to detect chromosomal point mutations linked to resistance.

### Data management

Registration of flock, house, age, weight, macroscopic lesions, and bacterial growth was conducted at animal level, whilst flock productivity and mortality data were provided by DanHatch Denmark A/S upon request.

## Results

### Study population

A total of 340 broiler breeders were collected. Farm A contributed with 140 birds, whilst Farm B and C delivered 96 and 104, respectively. From Farm A, nine birds (14.3%) aged 27–34 weeks were marked as putative healthy, i.e., controls, with this number being 14 (18.2%) in the birds aged 45–60 weeks. Farm B delivered three (7%) control birds in the young age category (27–34 weeks) and nine (17%) in the 45–60 weeks category. From Farm C, birds marked as healthy were only received among the older (45–60) birds (*n* = 9 (15.5%)).

Fifty-four broilers, hatched from eggs laid on Farm A aged 27–34 weeks, were collected, whilst this number was 52 and 48 from Farms B and C, respectively.

None of the flocks included in this study received any antibiotic treatment. Tables 1 and 2 present an overview of the study population, mortality, and productivity.

**Table 1:** **Overview of mortality, productivity, and the study population of broiler breeders**

General			Flock mortality		Productivity						Birds included in the study		
Farm	Flock size	Bird/m^2^	Mortality, total (%)	Total no. of dead birds	Eggs/hen	Hatching (%)	No. of chicks delivered/ hen	Chick mortality, first week (%)	Chick mortality, total, average (%)	Chick slaughter condemnation, average (%)	No. of birds for post-mortem examination	No. of birds sampled for bacteriology	No. of *Escherichia coli* isolates subjected to genome sequencing
Farm A	45,759	6.65	8.21	3757	168	86.7	135.3	0.91	2.90	1.50	140	120	22
Farm B	23,487	6.74	5.12	1203	173	86.7	144.1	0.79	2.88	1.34	96	74	10
Farm C	39,383	6.68	7.35	2895	174	86.7	144.7	0.86	3.66	1.33	104	87	17

**Table 2:** **Overview of the mortality, productivity, and study population of the progeny**

General			Flock mortality			Productivity			Birds included in the study		
Farm	Flock size	Bird/m^2^	Mortality, total (%)	Mortality, first week (%)	Total no. of dead birds	Feed conversion	Slaughter weight, average (g)	Slaughter condemnation (%)	Total no. of birds for post-mortem examination	No. of birds sampled for bacteriology	No. of *Escherichia coli* isolates subjected to genome sequencing
Progeny A	31,700	41.2	1.50	0.55	476	1.50	2140	0.7	54	54	5
Progeny B	27,000	41.7	1.31	0.52	415	1.47	2193	0.6	52	51	11
Progeny C	30,800	40.2	1.84	0.42	583	1.52	2148	0.6	48	48	12

### Post-mortem findings

A comprehensive overview of the lesions in the broiler breeders is presented in Table 3, whereas the gross pathology identified in the progeny is presented in Table 4.

**Table 3:** **Overview of gross lesions in broiler breeders from Farms A, B and C**

	Farm A (*n* = 140)			Farm B (*n* = 96)			Farm C (*n* = 104)			Total (*n* = 340)		
	27–34 weeks (*n* = 63)	45–60 weeks (*n* = 77)	Total	27–34 weeks (*n* = 43)	45–60 weeks (*n* = 53)	Total	27–34 weeks (*n* = 46)	45–60 weeks (*n* = 58)	Total	27–34 weeks (*n* = 152)	45–60 weeks (*n* = 188)	Total
General												
Euthanised	34 (54%)	52 (67.5%)	86 (61.4%)	23 (53.5%)	21 (39.6%)	44 (45.8%)	10 (21.7%)	21 (36.2%)	31 (29.8%)	67 (44.1%)	94 (50%)	161 (47.4%)
BW (kg)	3.3 ± 0.5	3.3 ± 0.8	3.3 ± 0.6	3.0 ± 0.6	3.2 ± 0.8	3.1 ± 0.7	2.8 ± 0.6	3.4 ± 0.8	3.1 ± 0.8	3.1 ± 0.6	3.3 ± 0.8	3.2 ± 0.7
No. of males	0 (0%)	4 (5.2%)	4 (2.9%)	0 (0%)	0 (0%)	0 (0%)	3 (6.5%)	3 (5.2%)	6 (5.8%)	3 (2%)	7 (3.7%)	10 (2.9%)
Dehydration	19 (30.2%)	24 (31.2%)	43 (30.7%)	9 (20.9%)	27 (50.9%)	36 (37.5%)	15 (32.6%)	25 (43.1%)	40 (35.5%)	43 (28.3%)	76 (40.4%)	119 (35%)
Emaciation	2 (3.2%)	3 (3.9%)	5 (3.6%)	2 (4.7%)	5 (9.4%)	7 (7.3%)	7 (15.2%)	2 (3.4%)	9 (8.7%)	11 (7.2%)	10 (5.3%)	21 (6.2%)
Skin, subcutis and foot pads												
Laceration (mating injuries)^a^	17 (27%)	19 (25.7%)	36 (25.7%)	10 (23.3%)	3 (5.7%)	13 (13.5%)	2 (4.3%)	2 (3.4%)	4 (3.8%)	29 (19.1%)	24 (12.8%)	53 (15.6%)
Bumblefoot^b^	1 (1.6%)	3 (3.9%)	4 (2.9%)	0 (0%)	2 (3.8%)	2 (2.1%)	1 (2.2%)	0 (0%)	1 (1%)	2 (1.3%)	5 (2.7%)	7 (2.1%)
Pododermatitis^c^	40 (63.5%)	23 (29.9%)	63 (45%)	13 (30.2%)	17 (32.1%)	30 (31.3%)	22 (47.8%)	36 (62.1%)	58 (55.8%)	75 (49.3%)	76 (40.4%)	151 (44.4%)
Bursitis presternalis	8 (12.7%)	8 (10.4%)	16 (11.4%)	15 (34.9%)	14 (26.4%)	29 (30.2%)	6 (13%)	14 (24.1%)	20 (19.2%)	29 (19.1%)	36 (19.1%)	65 (19.1%)
Skeletal system and joints												
Fracture (long bones)	1 (1.6%)	4 (5.2%)	5 (3.1%)	0 (0%)	4 (7.5%)	4 (4.2%)	0 (0%)	2 (3.4%)	2 (1.9%)	1 (0.7%)	10 (5.3%)	11 (3.2%)
Arthritis^d^	2 (3.2%)	6 (7.8%)	8 (5.7%)	3 (7%)	7 (13.2%)	10 (10.4%)	8 (17.4%)	2 (3.4%)	10 (9.6%)	13 (8.5%)	15 (8%)	28 (8.2%)
Sternal fracture(s)												
Total	2 (3.2%)	25 (32.5%)	27 (19.3%)	4 (9.3%)	31 (58.5%)	35 (36.5%)	2 (4.3%)	29 (50%)	31 (29.8%)	8 (5.3%)	85 (45.2%)	93 (27.4%)
0	61 (96.8%)	52 (67.5%)	113 (80.7%)	38 (88.4%)	22 (41.5%)	60 (62.5%)	45 (97.8%)	28 (48.3%)	73 (70.2%)	144 (94.7%)	102 (54.3%)	246 (72.4%)
1	2 (3.8%)	15 (19.5%)	17 (12.1%)	4 (9.3%)	19 (35.8%)	23 (24%)	2 (4.3%)	18 (31%)	20 (19.2%)	8 (5.3%)	52 (27.7%)	60 (17.6%)
2	0 (0%)	6 (7.8%)	6 (4.3%)	0 (0%)	4 (7.5%)	4 (4.2%)	0 (0%)	7 (12.1%)	7 (6.7%)	0 (0%)	17 (9%)	17 (5%)
3	0 (0%)	3 (3.9%)	3 (2.1%)	0 (0%)	6 (11.3%)	6 (6.3%)	0 (0%)	3 (5.2%)	3 (2.9%)	0 (0%)	12 (6.4%)	12 (3.5%)
> 4	0 (0%)	1 (1.3%)	1 (0.7%)	0 (0%)	2 (3.8%)	2 (2.1%)	0 (0%)	1 (1.7%)	1 (1%)	0 (0%)	4 (2.1%)	4 (1.2%)
Respiratory system												
Tracheal changes^e^	21 (33.3%)	18 (23.4%)	39 (27.9%)	11 (25.6%)	9 (17%)	20 (20.8%)	22 (47.9%)	23 (39.7%)	45 (43.3%)	54 (35.5%)	50 (26.6%)	104 (30.6%)
Pulmonary changes^f^	25 (39.7%)	27 (35.1%)	52 (37.1%)	16 (37.2%)	18 (34%)	34 (35.4%)	21 (45.7%)	22 (37.9%)	43 (41.3%)	62 (40.8%)	67 (35.6%)	129 (38%)
Airsacculitis^g^	14 (22.2%)	14 (18.2%)	28 (20%)	6 (14%)	4 (7.5%)	10 (10.4%)	12 (26.1%)	13 (22.4%)	25 (24%)	32 (21.1%)	31 (16.5%)	63 (18.5%)
Coelomic cavity												
Pericarditis^h^	6 (9.5%)	3 (3.9%)	9 (6.4%)	1 (2.3%)	2 (3.8%)	3 (3.1%)	0 (0%)	0 (0%)	0 (0%)	7 (4.6%)	5 (2.7%)	12 (3.5%)
Perihepatitis^h^	6 (9.5%)	3 (3.9%)	9 (6.4%)	3 (7%)	1 (1.9%)	4 (4.2%)	0 (0%)	0 (0%)	0 (0%)	9 (5.9%)	4 (2.1%)	13 (3.8%)
Fatty liver	15 (23.8%)	9 (11.7%)	24 (17.1%)	5 (11.6%)	5 (9.4%)	10 (10.4%)	0 (0%)	5 (8.6%)	5 (4.8%)	20 (13.2%)	19 (10.1%)	39 (11.5%)
Hepatic rupture	2 (3.2%)	2 (2.6%)	4 (2.9%)	0 (0%)	3 (5.7%)	3 (3.1%)	1 (2.2%)	5 (8.6%)	6 (5.8%)	3 (2%)	10 (5.3%)	13 (3.8%)
Peritonitis^h^	23 (36.5%)	28 (36.4%)	51 (36.4%)	10 (23.3%)	15 (28.3%)	25 (26%)	11 (23.9%)	19 (32.8%)	30 (28.8%)	44 (28.4%)	62 (33%)	106 (31.2%)
Renal changes^i^	17 (27%)	24 (31.2%)	41 (29.3%)	11 (25.6%)	19 (35.8%)	30 (31.3%)	27 (58.7%)	23 (39.7%)	50 (48.1%)	55 (36.2%)	66 (35.1%)	121 (35.6%)
Reproductive system												
In lay^j^	39 (61.9%)	20 (27.4%)	59 (43.4%)	16 (37.2%)	21 (39.6%)	37 (38.5%)	12 (27.9%)	27 (49.1%)	39 (39.8%)	67 (44.1%)	68 (36.2%)	135 (40.9%)
Egg bound	0 (0%)	0 (0%)	0 (0%)	0 (0%)	2 (3.8%)	2 (2.1%)	3 (7%)	1 (1.8%)	4 (4.1%)	3 (2%)	3 (1.6%)	6 (1.8%)
Perioophoritis^h^	8 (12.7%)	17 (23.3%)	25 (18.4%)	4 (9.3%)	12 (22.6%)	16 (16.7%)	5 (11.6%)	10 (18.2%)	15 (15.3%)	17 (11.9%)	39 (20.7%)	56 (17%)
Ovarian regression^k^	14 (22.2%)	28 (38.4%)	42 (30.9%)	15 (34.9%)	21 (39.6%)	36 (37.5%)	20 (46.5%)	28 (50.9%)	48 (49%)	49 (32.2%)	77 (41%)	126 (38.2%)
Salpingitis^l^	14 (22.2%)	10 (13.7%)	24 (17.6%)	8 (18.6%)	10 (18.9%)	18 (18.8%)	5 (11.6%)	9 (16.4%)	14 (14.3%)	27 (17.8%)	29 (15.4%)	56 (17%)
Regressed salpinx^m^	6 (9.5%)	16 (21.9%)	22 (16.2%)	7 (16.3%)	18 (34%)	25 (26%)	15 (34.9%)	20 (36.4%)	35 (35.7%)	28 (18.4%)	54 (28.7%)	82 (24.8%)
Cystic right oviduct/reminiscence	9 (14.3%)	14 (19.2%)	23 (16.9%)	8 (18.6%)	17 (32.1%)	25 (26%)	0 (0%)	6 (10.9%)	6 (6.1%)	17 (11.2%)	37 (19.7%)	54 (16.4%)
Miscellaneous												
Cannibalism	4 (6.3%)	3 (3.9%)	7 (5%)	0 (0%)	1 (1.9%)	1 (1%)	1 (2.2%)	0 (0%)	1 (1%)	5 (3.3%)	4 (2.1%)	9 (2.6%)
Neoplasia	1 (1.6%)	0 (0%)	1 (0.7%)	0 (0%)	3 (5.7%)	3 (3.1%)	0 (0%)	0 (0%)	0 (0%)	1 (0.7%)	3 (1.6%)	4 (1.2%)

Continuous data is presented with mean ± standard deviation

BW, bodyweight; n, number

^a^Caudolaterodorsally located lacerations involving skin and muscle often associated with substantial tissue necrosis, “pocket formation” and fibrinopurulent cellulitis

^b^Pododermatitis with profound swelling due to purulent material and/or fibrosis

^c^Footpad lesions consisting of discoloration, hyperkeratosis and/or ulceration

^d^Exudate present within any of the major joints

^e^Presence of hyperaemia, haemorrhage, exudate, or excessive amounts of mucus with a turbid appearance

^f^Oedema, consolidation and/or exudate

^g^Presence of fibrinous, purulent or fibrinopurulent exudate and/or thickening and opaqueness of the air sac

^h^Presence of fibrinous, purulent or fibrinopurulent exudate or fibrotic repair

^i^Increased tubular pattern, urate deposition in ureters and/or swelling of the kidneys

^j^In lay defined as hens having a developing- or fully developed egg present within the salpinx

^k^Complete absence of mature follicles or presence of atretic follicles indicating an early state of regression

^l^Presence of fibrinous, purulent, fibrinopurulent or mucopurulent exudate

^m^Pale salpinx containing no developing egg and being less than half the expected size

**Table 4:** **Overview of gross lesions in broilers from Progenies A, B and C**

	Progeny A (*n* = 54)	Progeny B (*n* = 52)	Progeny C (*n* = 48)	Total (*n* = 154)
General				
Euthanised	44 (81.5%)	47 (90.4%)	30 (62.5%)	121 (78.6%)
Age (days)				
Mean	8 ± 1.4	8.1 ± 2.4	6.1 ± 1.5	7.4 ± 2
Min	5	5	4	4
Median	8	8	7	7
Max	10	11	8	11
BW (g), day				
4	–	–	50 ± 19	50 ± 19
5	97 ± 27	65 ± 18	–	72 ± 24
6	156 ± 13	135 ± 10	80 ± 32	119 ± 39
7	133 ± 44	–	144 ± 48	140 ± 46
8	147 ± 55	193 ± 56	164 ± 20	166 ± 50
9	139 ± 61	–	–	139 ± 61
10	187 ± 91	193 ± 58	–	191 ± 68
11	–	253 ± 31	–	253 ± 31
Gender				
Male	22 (40.7%)	20 (38.5%)	19 (39.6%)	61 (39.6%)
Female	32 (59.3%)	32 (61.5%)	29 (60.4%)	93 (60.4%)
Dehydration	15 (27.8%)	12 (23.1%)	17 (35.4%)	44 (28.6%)
Peri-cloacal urate	2 (3.7%)	6 (11.5%)	7 (14.6%)	15 (9.7%)
Umbilicus and yolk sac				
Unhealed umbilicus				
Total	14 (25%)	15 (28.8%)	23 (47.9%)	52 (33.8%)
Open	1 (1.9%)	0 (0%)	1 (2.1%)	2 (1.3%)
String	11 (20.4%)	13 (25%)	16 (33.3%)	40 (26%)
Button	2 (3.7%)	2 (3.8%)	6 (12.5%)	10 (6.5%)
Omphalitis^b^	3 (5.6%)	2 (3.8%)	7 (14.6%)	12 (7.8%)
Yolksacculitis^c^	4 (7.4%)	9 (17.3%)	11 (22.9%)	24 (15.6%)
Retained yolk sac^d^	3 (8.6%)	5 (15.6%)	1 (2.1%)	9 (11.8%)
Respiratory tract				
Tracheal changes^e^	3 (5.6%)	1 (1.9%)	2 (4.2%)	6 (3.9%)
Pulmonary changes^f^	38 (70.4%)	39 (75%)	34 (70.8%)	111 (72.1%)
Airsacculitis	3 (5.6%)	1 (1.9%)	1 (2.1%)	4 (2.6%)
Coelomic cavity				
Pericarditis^g^	3 (5.6%)	6 (11.5%)	6 (12.5%)	15 (9.7%)
Perihepatitis^g^	2 (3.7%)	2 (3.8%)	7 (14.6%)	11 (7.1%)
Peritonitis^g^	3 (5.6%)	5 (9.6%)	6 (12.5%)	14 (9.1%)
Ascites	4 (7.4%)	4 (7.7%)	2 (4.2%)	10 (6.5%)
Renal changes^h^	20 (37%)	14 (26.9%)	17 (35.4%)	51 (33.1%)
Gastrointestinal tract				
Feed in crop	51 (94.4%)	44 (84.6%)	33 (68.8%)	128 (83.1%)
Empty/sparse GI content	5 (9.3%)	8 (15.4%)	15 (31.3%)	28 (18.2%)
Ulcus ventriculi	1 (1.6%)	3 (5.8%)	5 (10.4%)	9 (5.8%)

Continuous data are presented with mean ± standard deviation

BW, bodyweight; GI, gastrointestinal; n, number

^a^Patent opening of the umbilicus without complications, or with entrapment of tissue forming either a string or a button

^b^Presence of hyperaemia and/or oedema in the umbilical area

^c^Alterations to the yolk consistency, e.g., watery, lumpy, thickened or inspissated yolk possibly with changes to the colour or odour as well as excessive hyperaemia

^d^Presence of the yolk sac in chickens older than seven days

^e^Presence of hyperaemia, haemorrhage, exudate, or excessive amounts of mucus with a turbid appearance

^f^Oedema, consolidation and/or exudate

^g^Presence of fibrinous, purulent or fibrinopurulent exudate or fibrotic repair

^h^Swelling of the kidneys and/or increased tubular pattern

^i^Presence of exudate

**Figure 1:**
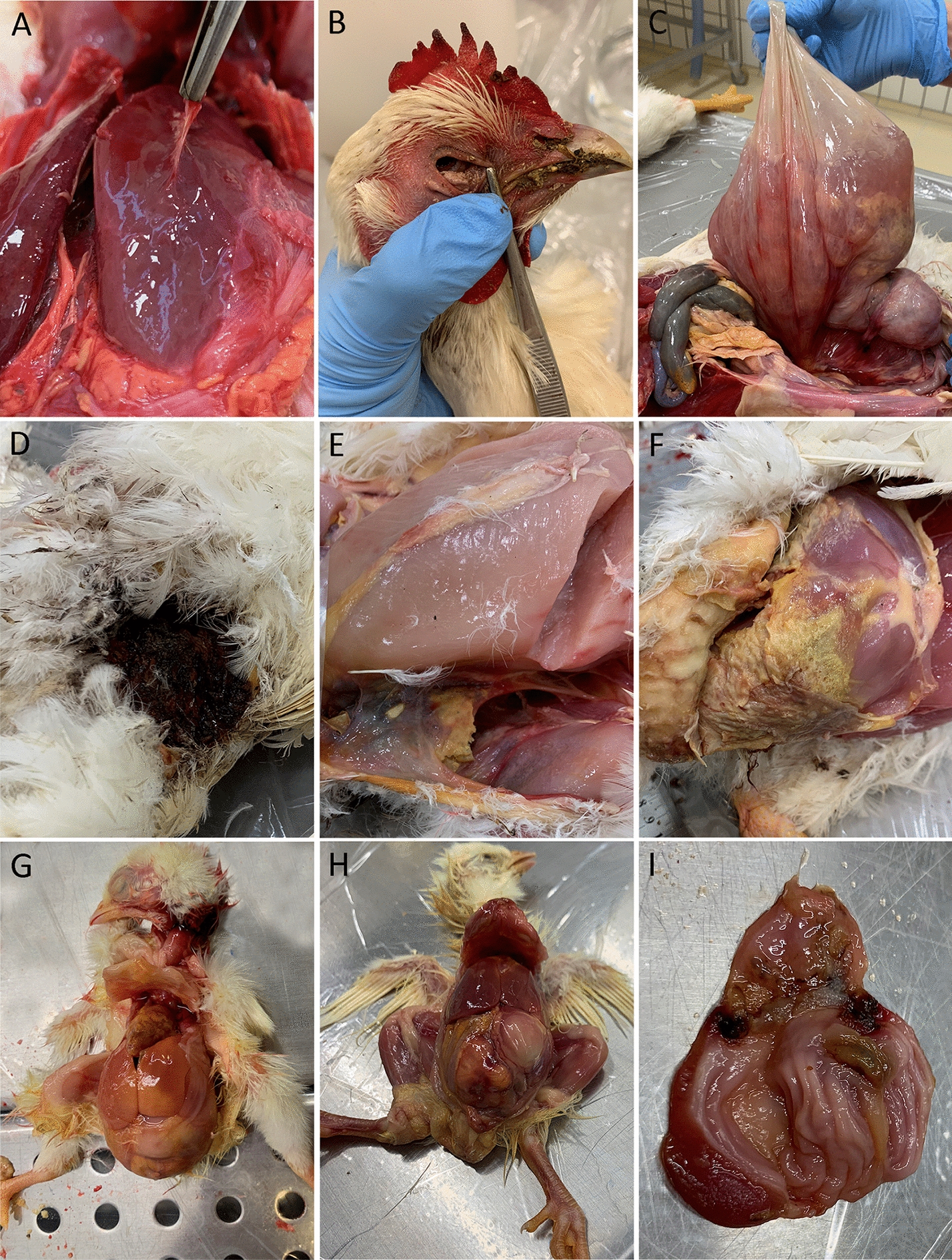
**Lesions observed at post-mortem examination.** **A** Chronic perihepatitis. The bird had multiple chronic lesions consistent with polyserositis, e.g., opaque and thickened thoracic air sacs, chronic adhesive pericarditis, and numerous adhesions between several parts of the intestines, the mesovarium and salpinx. The ovary of the hen was inactive. **B** Deposition of urate in the conjunctiva of a hen. Throughout the coelomic cavity and major joints urate deposition was present. The ureters were occluded  by urate and the kidneys were swollen and showed an increased tubular pattern. The ovary was in regression and no egg was present within the oviduct. **C** Massive cystic enlargement of the oviduct containing fluid with lumps of fibrinopurulent exudate. The ovary was inactive and the remaining coelomic organs were all cranially displaced. **D** A massive skin laceration with a caudolaterodorsal location consistent with a mating injury. Externally, the wound measured approximately 9 × 5 cm, with multiple internal pockets with presence of necrotic tissue, fibrinopurulent material and bedding. **E** Fibrinopurulent exudate extending from the wound in **D**. The hen was not in lay , and the ovary had completely regressed. **F** A lesion similar to **D**–**E** with fibrinopurulent cellulitis extending from the caudodorsal part of the bird to cranial part of the leg. The external measurement of the wound was approximately 5 × 8 cm. The bird was not in lay , and the ovary was in regression with a few atretic follicles present. Fibrinopurulent peritonitis was present. **G** Purulent peritonitis in a young chicken. The yolk sac appeared grossly normal though a “button” was present in the unhealed umbilicus, and the yolk sac and liver yielded pure growth of *E. coli*. **H** Hyperaemia of the yolk sac which contained a partly inspissated content (yolksacculitis). The umbilical area was hyperaemic (omphalitis) extending to the abdominal wall, fibrinous pericarditis and perihepatitis was present as well as fibrinopurulent airsacculitis and peritonitis. **I** Ulcus ventriculi in a young chicken adjacent to the gastric isthmus.

Briefly, exudative peritonitis was present in 31.2% of the birds with perihepatitis and pericarditis being considerably less represented in only 3.8% and 3.5%, respectively. In females, salpingitis and perioophoritis both occurred in 17% of the birds, yet without any strict tendency to necessarily occur simultaneously in the same bird. Airsacculitis were present in 18.5% of the hens. The fractured long bones (*n* = 11 equalling 3.2%) included eight femoral and three tibial bones. Four birds from Farm C had an acute fracture of the beak. Two of these had additional injuries to the head area with extensive subcutaneous haemorrhage. Four additional birds from Farm C had similar trauma to the head area without any damage to the beak. These lesion types were not represented in animals from Farms A and B. The presence of purulent, fibrinous or fibrinopurulent arthritis identified in 8.2% of the birds was almost exclusively restricted to the intertarsal joints (*n* = 26), with the hip joint accounting for only two cases of arthritis. In adult females, 15.6% of the collected birds had caudolaterodorsal lacerations (mating injuries) (Figures 1D–F) ranging in sizes from 2 to 20 cm in length (mean 7.5 ± 3.5 cm). All revealed varying degree of associated profound tissue damage in relation to the laceration, i.e., muscle necrosis, fibrinopurulent cellulitis and/or pocket formation, and the lesions often contained bedding material. Registration on the reproductive status revealed that 64.2% of the birds with lacerations were not in active lay. Pododermatitis lesions, defined as the presence of discolouration, hyperkeratosis and/or ulceration, ranged in size from 2 to 40 mm (mean 9.4 ± 7 mm). Only seven birds had additional profound swelling with purulent material and/or fibrosis, i.e., bumblefoot.

All birds submitted as presumed clinically healthy were females, and 86.4% were in active lay, whereas this was the case in 40.9% of the remaining hens. In the putative healthy birds, 43.8% were graded as obese, 38.6% as above average and 18.2% as having a normal body condition, whilst these figures were 16.9%, 31.8% and 36.4%, respectively, among the remaining birds. In the latter, 18.5% were graded as having a body condition below average and 6.2% were cachectic. Two (4.5%) of the putative healthy hens had purulent peritonitis, one (2.3%) had purulent perioophoritis, one (2.3%) purulent salpingitis, and six (13.6%) had pulmonary changes.

A total of 10 roosters were submitted to post-mortem examination, of which Farm A contributed with four and Farm C with six, and all were euthanised except two males from Farm C. One of these suffered from a complete intestinal obstruction caused by *Ascaridia galli*, whereas the other was cachectic and dehydrated with empty intestines and had bilateral hypoplasia of the kidneys. The remaining male birds showed various lesions (purulent arthritis, tibial fracture, deformity to a toe and foot pads most likely as a consequence of a healed fracture, chronic pododermatitis, acute fracture of the beak and necrosis of the comb).

Broilers gathered from Progeny C were generally younger than those from Progeny A and Progeny B, and seemed to excel on some parameters, e.g., unhealed umbilicus, omphalitis, perihepatitis, absence of feed within the crop and empty/sparse content in the gastrointestinal tract (Table 4).

### *Escherichia coli* diversity

**Figure 2:**
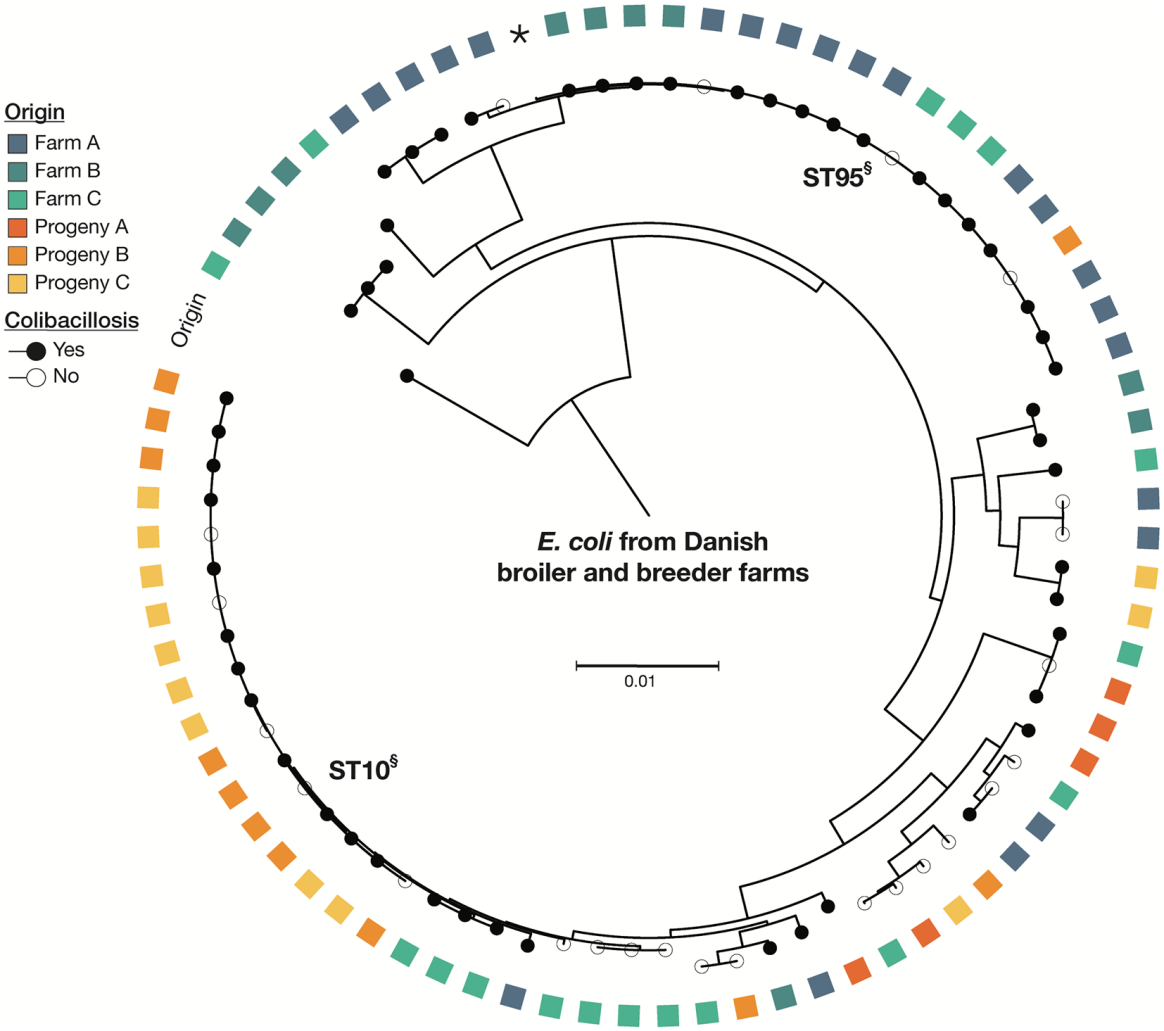
**Phylogenetic tree presenting *Escherichia coli* obtained during a non-outbreak period.** Midpoint rooted phylogenetic tree based on core genome single-nucleotide polymorphisms (SNPs) of the 77 *E. coli* isolated from Danish broiler breeder and broiler farms. Farm/progeny origin is indicated on the phylogeny, as is colibacillosis status of the host. Colibacillosis defined as presence of fibrinous-, purulent-, fibrinopurulent or mucopurulent exudate inflammation is presented with black circles, whereas the absence is denoted with open circles. §Single-locus variants are observed within the ST10 cluster (ST7321 (*n* = 3), ST6665 (*n* = 3) and ST1141 (*n* = 1)). The tree is based on 222 391 SNPs detected within a ~3.03 Mbp conserved core genome across the collection. *Depicts *E. coli* isolate E51 used as reference. Scale bar indicates substitutions per site. Among the 77 isolates, 24 were obtained from the liver, 16 from the salpinx, 22 from the lung, and 12 from the yolk sac. The spleen, umbilical area, and a case of arthritis each contributed with a single isolate. Isolates from birds with an absence of lesions consistent with colibacillosis represented isolation sites: liver, lung, salpinx, yolk sac and joint (*n* = 6, 11, 2, 4 and 1, respectively).

In total, 77 *E. coli* isolates obtained from extraintestinal organs in birds during a non-outbreak situation were selected for sequencing. This revealed a diverse range of sequence types (*n* = 23) (Table 5 and Figure 2). Overall, ST95 and ST10 were the most prevalent sequence types (27.3% and 23.4%, respectively) and birds lacking signs of colibacillosis were represented among both these sequence types.

**Table 5:** **Characteristics of the detected sequence types**

Sequence type	Prevalence within farm^a^	No. of positive birds/farm	Total no	Serotype(s)	SNP-distance within farm^b^	SNP-distance between farms
ST10	Farm A1 (4.5%)	1	18 (23.4%)	O71:H40, –:H40	–	0–7038
	Farm C3 (17.6%)	2			7	
	Progeny B5 (45.5%)	4			1–960	
	Progeny C9 (75%)	7			0–959	
ST23	Farm C1 (5.9%)	1	1 (1.3%)	O78:H17	–	–
ST38	Farm C1 (5.9%)	1	1 (1.3%)	O7:H15	–	–
ST58	Progeny C1 (8.3%)	1	1 (1.3%)	O86:H30	–	–
ST69	Farm B2 (20%)	2	2 (2.6%)	O15:H6	1861	–
ST95	Farm A13 (59.1%)	11	21 (27.3%)	O2:H5, O1:H7, -:H5	0–3691	0–3691
	Farm B 4 (40%)	2			39	
	Farm C3 (17.6%)	3			0–1	
	Progeny B1 (9.1%)	1			–	
ST117	Farm C1 (5.9%)	1	1 (1.3%)	-:H4	–	–
ST155	Farm C1 (5.9%)	1	2 (2.6%)	O8:H20, O37:H10	–	2526
	Progeny A1 (20%)	1				
ST219	Farm C1 (5.9%)	1	3 (3.9%)	O138:H48, –:H48	–	29–30
	Progeny A2 (40%)	2			1	
ST362	Farm A2 (9.1%)	1	2 (2.6%)	O7:H6	–	–
ST442	Progeny B1 (9.1%)	1	1 (1.3%)	O91:H21	–	–
ST457	Farm B3 (30%)	1	3 (3.9%)	O11:H25	–	–
ST746	Farm B1 (10%)	1	3 (3.9%)	O100:H30, –:H18	–	10–7545
	Farm C1 (5.9%)	1			–	
	Progeny B1 (9.1%)	1			–	
ST770	Farm C1 (5.9%)	1	1 (1.3%)	O15:H16	–	–
ST1141	Farm C1 (5.9%)	1	1 (1.3%)	O113:H4	–	–
ST1426	Progeny A1 (20%)	1	1 (1.3%)	O8:H16	–	–
ST1564	Farm A1 (4.5%)	1	1 (1.3%)	–:H21	–	–
ST1882	Progeny C2 (16.7%)	2	2	O23:H4, –:H4	0	–
ST3270	Progeny A1 (20%)	1	1 (1.3%)	O8:H16	–	–
ST6665	Farm C3 (17.6%)	1	3 (3.9%)	O8:H30	–	–
ST7321	Progeny B3 (27.8%)	2	3 (3.9%)	O71:H40	0	–
ST7614	Farm A2 (9.1%)	2	2 (2.6%)	O9:H19	4	–
ST11774	Farm A3 (13.6%)	3	3 (3.9%)	O46:H31, –:H31	1–4	–

SNP, single nucleotide polymorphism; ST, sequence type

^a^Only farms positive for the given ST are listed

^b^Only between bird comparison

In four birds, contributing with multiple isolates obtained from different organs, different sequence types were found depending on the organ of isolation. There was no tendency of a particular organ to differ more from the rest and the birds were all classified as having colibacillosis. The variation between isolates of similar sequence type, isolated from the same bird, showed a SNP-distance of 0–2 across a core genome of 58% (3.03 Mb). From a single bird, *E. coli* isolated from the magnum was sequenced twice (different colonies) revealing a single SNP between the two colonies. Curiously, when comparing the ST95 isolates from Progeny B to Farm B, a variation between 14 and 39 SNPs was observed, whereas the SNP-based distances from Progeny B to Farm A and Farm C were between 0-3686 and 10–11, respectively. Likewise, comparing the diversity among the ST10 isolates from Farm C and Progeny C, the SNP-distances were between 4419 and 4428, whilst the SNP-distance between Progeny B and Progeny C varied from 0 to 958, which was comparable with the within-farm variation on the farms. For ST746, the genetic diversity was larger between Farm B and Progeny B than between Farm C and Progeny B with 7545 versus 10 SNPs, respectively. Overall, the presence of resistance markers was low with *mdfA* and *sitABCD* (multidrug transporter and hydrogen peroxide resistance, respectively) being the primary findings, except in one isolate (ST1564) additionally containing *bla*_*TEM1B*_, *sul2*, *drfA1*, *tet(A)*, *aadA1*, and *qnrS1*, thereby, conferring resistance towards beta-lactams, sulphonamides, trimethoprim, tetracycline, aminoglycosides, and quinolones, respectively. Resistance towards quinolones was detected in one more isolate (ST770) containing the *parE* (I355T) mutation. Generally, ST95 seemed to harbour *sul2* to a great extent with 19/21 carrying this gene. Detailed information on resistance markers is available in Additional file 3.

## Discussion

In the present study, a comprehensive characterisation of lesions within three broiler breeder farms, as well as related progeny, all having acceptable levels of background mortality, is presented. Furthermore, *E. coli* isolates obtained from the farms were characterised by WGS.

All farms exhibited exemplary production results with an absence of general disease problems and a low mortality confirming a non-outbreak status. A wide range of infection-related lesions was registered within the broiler breeders, with peritonitis being the most prevalent, whereas yolksacculitis was the most prominent in the broilers. Both lesion-types are characteristic of *E. coli* infection [1, 5, 32].

Mating injuries were a common finding among the hens and must be considered a possible portal of entrance for bacteria such as *E. coli*. These lacerations also represent a major welfare concern [33] as the lesions are of considerable size and often associated with severe damage to the surrounding tissues. The lower level of mating injuries in hens from Farm C is most likely attributable to farm management on this location, as animals exhibiting this lesion-type are only rarely culled due to a belief in their ability to produce eggs despite their injuries (personal communication). Yet, in the current study, it was revealed that the majority of these birds were not in active lay.

Pododermatitis is another well-known factor impairing the welfare of poultry [34], and especially in broilers, footpad lesions have received marked attention and is routinely evaluated upon slaughter and used as a welfare parameter [34, 35]. However, in broiler breeders, less is known about the prevalence of footpad lesions [1]. An increased frequency as the birds ages has been reported previously but was not apparent throughout the farms in the current study [1, 36]. The prevalence both differed between farms and time periods, likely reflecting the condition of the bedding material— a well-known risk factor [34, 36, 37]. The occurrence of sternal fractures increased with age, which is in accordance with previous reports [38], and seemed to differ in prevalence between the farms in the current study.

A total of 23 different sequence types were represented among the 77 sequenced *E. coli* isolates. Among these, 16 different O-antigens were identified, including O78, O1 and O2 commonly associated with APEC [39, 40], whilst 17 different H-antigens were demonstrated. In the Danish broiler breeder production, a vaccine strategy targeting *E. coli* was implemented in 2016 following a devastating clonal *E. coli* outbreak [22]. The strategy was based on the commercial vaccine Poulvac^®^ *E. coli* and an autogenous vaccine containing the outbreak isolates ST140 O1:H7 (clonal complex 95) and ST117 O78:H4 with subsequent modifications to the vaccine rendering broiler breeders in the current study to be vaccinated with ST117 O78:H4, ST117 O53:H4 and ST95 O1:H7 (personal communication). Prior to vaccine implementation, particularly ST117 had been identified as the primary agent in the aforementioned Danish outbreak [15]. Intriguingly, ST117 was only isolated from a single adult hen in the present study despite its former role as a major contributor to *E. coli*-related mortality in Denmark [15]. Though it is tempting to contribute this almost negligible occurrence of ST117 to the effects of the autogenous vaccine—especially considering the support from recent studies of their protective effect and ability to alter the *E. coli* population on farms [41–43], the relative absence of ST117 might simply reflect the study population, as the current study presents sporadic cases of colibacillosis on non-outbreak farms and, therefore, could be dominated by opportunistic *E. coli* strains and not “true” APEC.

On the contrary to ST117, ST95, likewise known to have previously been a prominent sequence type involved in outbreaks of colibacillosis [18, 44], was greatly represented amongst the broiler breeders but only occurred in a single isolate from the progeny. In fact, a dissimilarity between sequence types prevalent in the adult broiler breeders suffering from colibacillosis and the types found in their progeny flocks was apparent, with ST10 being a main finding within the broilers. A noteworthy finding is also the presence of both ST95 and ST10 in birds without lesions consistent with colibacillosis, suggesting the ability to cope with their extraintestinal presence under some circumstances. This could indicate a lack of a contributing factor in these animals, e.g., stress or comorbidities providing an opportunity to act as a pathogen.

Interestingly, isolates from epidemiologically linked parent and progeny farms were not necessarily more genetically similar than those from unrelated farms. Also, in several cases, there were an absence or only a few SNPs between isolates from unrelated farms, and the within-farm variation and between-farm variation were often similar, thus indicating a common or recent source across farms.

Generally, the occurrence of resistance markers was low especially within isolates obtained from the progeny, and the findings were comparable to those previously reported in Denmark [45, 46]. Among ST95, the sequence type mainly obtained from broiler breeders, the presence of the *sul2* gene entailing resistance towards sulphonamides was common. This finding corresponds to previous observations in Danish broiler breeders, likewise reporting ST95 to often contain genes linked to sulphonamide resistance [47]. A single isolate exhibited multidrug-resistance with a presence of genes conferring resistance towards beta-lactams, sulphonamides, trimethoprim, tetracycline, aminoglycosides, and quinolones.

In conclusion, a comprehensive insight into the occurrence of lesions in broiler breeders and young broilers from farms without general disease problems is for the first time presented together with whole-genome sequencing of *E. coli* isolates. This revealed a diverse collection of *E. coli*, including several sequence types previously described as APEC, which could even be isolated from birds lacking colibacillosis-like lesions. The study emphasizes the elusive nature of the APEC pathotype and the great diversity of *E. coli* isolated from extraintestinal sites in poultry during non-outbreak situations.

## Supplementary Information

**Additional file 1.** **Standardised necropsy scheme for broiler breeders**.

**Additional file 2.** **Standardised necropsy scheme for broilers**.

**Additional file 3.** **Phylogenetic tree with presentation of resistance markers**.

## Data Availability

Data, upon which the conclusions in this manuscript relies, are presented within the paper and the supplementary material. Bacterial isolates and whole genome sequences are available from the corresponding author upon request.
