# Supplementary material for: Longitudinal study on background lesions in broiler breeder flocks and their progeny, and genomic characterisation of Escherichia coli
Source: Vet Res. 2022 Jul 7;53:52. doi: 10.1186/s13567-022-01064-7 (PMC9264609; doi:10.1186/s13567-022-01064-7)
Supplement: Supplementary file 1 — Additional file 1. Standardised necropsy scheme for broiler breeders. [file 13567_2022_1064_MOESM1_ESM.pdf]

Farm:

Journal #:

Date:

Age:

Weeks ____ Days ____

Comments:

								**ID #**
								**Farm #**
								**Age #**
								**House #**
								**Euthanasia** (y / n)
								**Date of death**
								**BW (kg)**
								**Gender**
								**Crop** (empty / content present / filled)
								**Other** (Cadavarotitas (mild / pronounced +) / cyanose / other)
								**Skin and plumage** (NC / lesions / naked areas. / "hanetråd")
								**Natural orifices** (NC / urate / faeces)
								**Food pads** (NC / discoloration / hyperkeratosis (mm) / ulceration (mm) / profundal swelling)
								**Sinus infraorbitalis** (NC / serous fluid / purulent / fibrinopurulent)
								**Conjunctivas** (hyperaemia / oedema)
								**Mucus membranes** (NC / dark / hyperaemia / smudged / cyanosis)
								**Esophagus / oral cavity – feed** (y / n / other)
								**Trachea** (NC / hyperaemia / mucus / haemorrhagic / other)
								**Larynx** (NC / hyperaemia / mucus / haemorrhagic / other)
								**Dehydrated** (y / n)
								**BCS** (cachetic -- / below average - / NC / Above average + / obese ++)
								**Dark muscles** (y / n)
								**Subcutis** (NC / dark / hyperaemic / oedema)
								**Art. coxae –** FHN (head/collum/cartilage) **inflamatio** (serous / fibrinous / purulent / fibrinopurulent)
								**Art. genus** (serous / fibrinous / purulent / fibrinopurulent)
								**Art. intertarsalis** (serous / fibrinous / purulent / fibrinopurulent)
								**Peritoneum** (serous / fibrinous / purulent / fibrinopurulent)
								**Sternal fracture** y (#) / n
								**Liver** (NC / enlarged / increased texture / fragile / pale / fibrinous / fibronopurulent / purulent)
								**Spleen** (NC / enlarged distended / flaccid)
								**GI** (hyperaemia / congestion)
								**In lay** (y / n) (developing egg / fully developed egg)
								**Follicles** (Juvenile / active / flaccid (atretic) / regression / hyperaemia / inactive / oophritis (fibrinous / fibrinopurulent)
								**Salpinx** (Juvenile / active (size) / oedema) / hyperaemia / inactive / salpingitis (fibrinous / fibrinopurulent / mucus) / congested / inactive / pale / atrophic)
								**Right oviduct** (cyst / reminiscence)
								**Pericardial sac** (NC / opque / serous / fibrinous / purulent / fibrinopurulent)
								**Pericardial fat** (NC / petechiae / ecchymosis / oedema)
								**Cardiac muscle** (NC / epicardial fibrinopurulent layer / oedema / congestion / pale)
								**Lungs** (NC / oedema / congestion / fibrinopurulent exudate (layer / bronchopneumonia)
								**Air-sacs** (NC / opaque / fibrinous / purulent / fibrinopurulent)
								**Kidney** (NC / swollen / increased tubular pattern)
								**Comments**
