# Supplementary material for: Longitudinal study on background lesions in broiler breeder flocks and their progeny, and genomic characterisation of Escherichia coli
Source: Vet Res. 2022 Jul 7;53:52. doi: 10.1186/s13567-022-01064-7 (PMC9264609; doi:10.1186/s13567-022-01064-7)
Supplement: Supplementary file 2 — Additional file 2. Standardised necropsy scheme for broilers. [file 13567_2022_1064_MOESM2_ESM.pdf]

Farm:

Age:

Name (writer):

Date:

Comments:

ID #										
Euthanasia<br>*(y / n)*										
Date of death										
Age (days)										
House										
Parent flock										
Age parents (weeks)										
BW (gram)										
Crop<br>*(empty / content present / filled (bedding / feed))*										
Skin and plumage<br>*(NC / lesions / naked areas)*										
Hock joint cutis<br>*Redness (y / n)*										
Navel area<br>*(NC / swelling / hyperaemia / open / drumstick / button)*										
Natural orifices<br>*(NC / urate / faeces)*										
Prominent vein<br>*(y / n)*										
Smudged head area<br>*(y / n)*										
Dehydrated<br>*(y / n)*										
Sinus infraorbitalis<br>*(serous fluid / purulent / fibrinopurulent)*										
Conjunctiva / eye<br>*(hyperaemia / oedema / exudate (f / fp))*										
Esophagus / oral cavity<br>*(y / n)*<br>*(feed / bedding)*										
Mucus membranes<br>*(dark / hyperaemia / smudged / cyanosis)*										
Dark muscles<br>*(y / n)*										
Subcutis<br>*(NC / dark / hyperaemic / oedema)*										
Abdominal wall<br>*(NC / vessel injection)*										
Art. coxae<br>*Swollen (y / n)*<br>*FHN (head/collum/cartilage)*<br>*(serous / serohaemorr. / fibrinous / purulent / fibrinopurulent)*										
Art. Genus<br>*Swollen (y / n)*<br>*(serous / fibrinous / purulent / fibrinopurulent)*										
Art. Intertarsalis<br>*Swollen (y / n)*<br>*(serous / fibrinous / purulent / fibrinopurulent)*										
Yolk-sac<br>*(y / n)*<br>*(hyperaemia / congestion / fibrin / purulent / fibrinopurulent)*<br>*Content (liquid / dry)*										
Air-sacs<br>*(fibrinous / purulent / fibrinopurulent)*										
Pericardial sac<br>*(fibrinous / purulent / fibrinopurulent)*										
Liver<br>*(serous / fibrinous / purulent / fibrinopurulent)*										
Peritoneum<br>*(serous / fibrinous / purulent / fibrinopurulent)*										
Reactive spleen<br>*(y / n)*										
Content in GI<br>*(y / n)*<br>*(feed / bedding)*										
Ventriculus<br>*(NC / erosion / ulceration)*<br>*Colour (white / yellow-brown)*										
Kidney<br>*(NC / swollen / increased tubular pattern / urate)*										
Gender<br>*(m / f)*										
Lungs<br>*(oedema / consolidation)*										
Comments										
