# Supplementary figures and images for: Longitudinal study on background lesions in broiler breeder flocks and their progeny, and genomic characterisation of Escherichia coli
Source: Vet Res. 2022 Jul 7;53:52. doi: 10.1186/s13567-022-01064-7 (PMC9264609; doi:10.1186/s13567-022-01064-7)

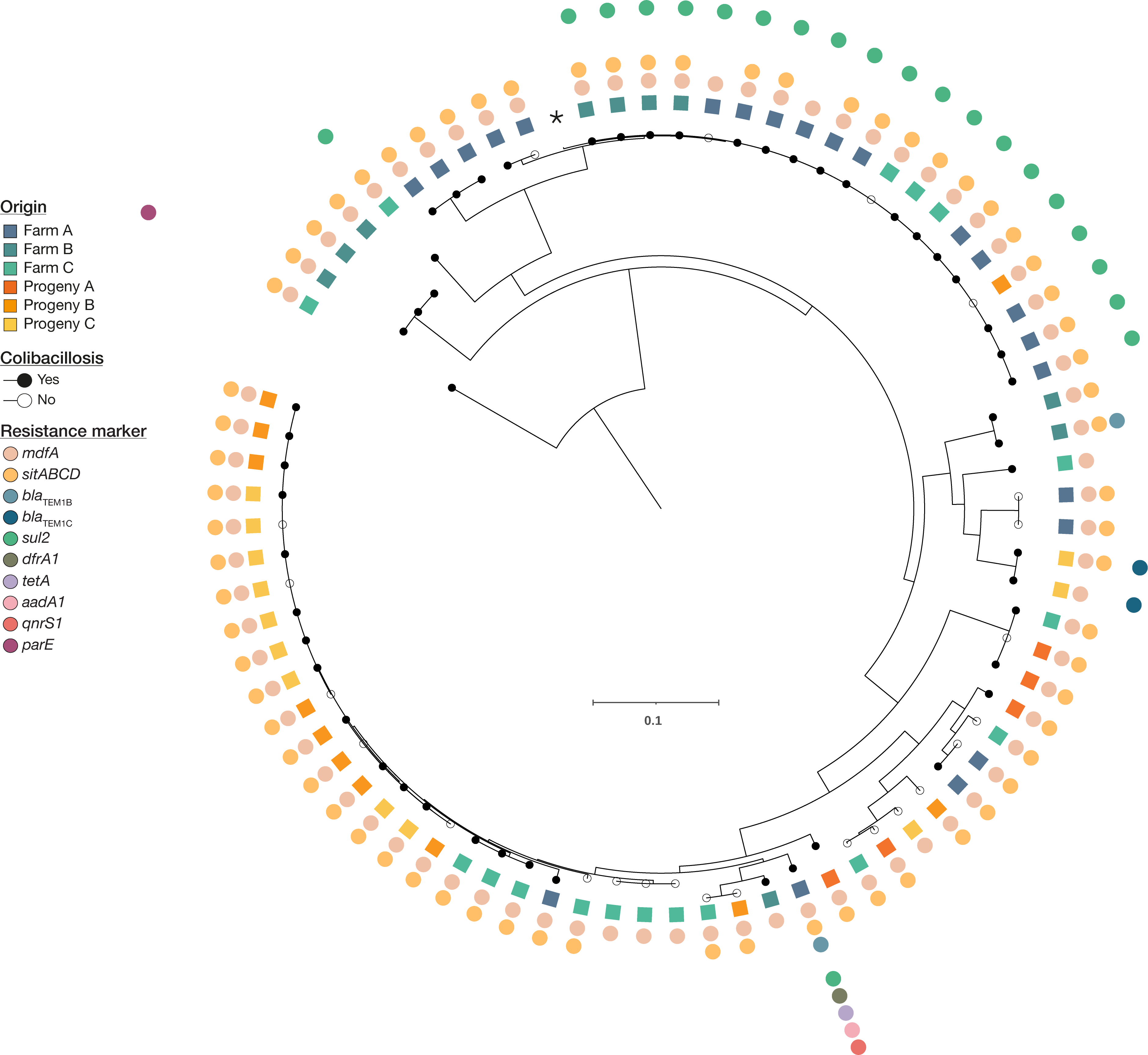

Supplement: Supplementary file 3 — Additional file 3. Phylogenetic tree with presentation of resistance markers. [file 13567_2022_1064_MOESM3_ESM.tif]
